# Deciphering Host–Virus Interactions and Advancing Therapeutics for Chronic Viral Infection

**DOI:** 10.3390/v17030390

**Published:** 2025-03-10

**Authors:** Majid Eslami, Neda Arjmand, Fatemeh Mahmoudian, Ali Babaeizad, Hamed Tahmasebi, Fahimeh Fattahi, Valentyn Oksenych

**Affiliations:** 1 Cancer Research Center, Semnan University of Medical Sciences, Semnan 35147-99442, Iran; m.eslami@semums.ac.ir (M.E.);; 2 Department of Bacteriology and Virology, Faculty of Medicine, Semnan University of Medical Sciences, Semnan 35147-99442, Iran; 3 Department of Obstetrics and Gynecology, Tehran Medical University, Tehran 14167-53955, Iran; 4 Student Research Committee, Semnan University of Medical Sciences, Semnan 35147-99442, Iran; 5 School of Medicine, Shahroud University of Medical Sciences, Shahroud 36147-73943, Iran; 6 Clinical Research Development Unit of Ayatollah-Khansari Hospital, Arak University of Medical Sciences, Arak 38186-49433, Iran; 7 Faculty of Medicine, University of Bergen, 5020 Bergen, Norway

**Keywords:** chronic infection, cytokine, host–virus interactions, immune system, viral evasion

## Abstract

Chronic viral infections like HIV, HBV, and HCV establish persistent interactions with the host immune system, resulting in immune evasion and long-term immune dysfunction. These viruses use a range of strategies to limit host defenses, such as downregulating MHC class I, disrupting interferon signaling, altering apoptosis pathways, and suppressing cytotoxic T-cell activity. Key viral proteins, including HIV Nef, HBV X protein, and HCV NS5A, interfere with antigen presentation and JAK/STAT signaling, thereby reducing antiviral immune responses. Chronic infections induce immune exhaustion due to persistent antigen exposure, which leads to the expression of inhibitory receptors like PD-1 and CTLA-4 on T cells. Viral epigenetic changes, such as N6-methyladenosine modifications and histone deacetylation, enhance immune evasion by modulating gene expression in infected cells. Viruses further manipulate host cytokine networks by promoting an immunosuppressive environment through IL-10 and TGF-β secretion, which suppress inflammatory responses and inhibit T-cell activation. This review examines the molecular/cellular mechanisms that enable chronic viruses to escape host immunity, focusing on antigenic variation, cytokine disruption, and control of apoptotic pathways. It also addresses how host genetic factors, such as HLA polymorphisms, influence disease progression. Lastly, we discuss host-targeted therapies, including immune checkpoint inhibitors, cytokine treatments, and CRISPR.

## 1. Mechanisms of Host–Virus Interaction in Chronic Viral Infections

Infections exist within a dynamic and metastable equilibrium. In acute infections, both the host and the virus undergo continual changes until the infection is resolved, the host succumbs to the disease, or the infection evolves into a chronic state [1,2]. These infections are characterized by the persistent presence of the virus in the host, which, in some cases, can lead to long-term health complications. Notable examples include human immunodeficiency viruses 1 and 2 (HIV-1 and HIV-2), which are responsible for acquired immune deficiency syndrome (AIDS). Additionally, the hepatitis C virus (HCV) and hepatitis B virus (HBV) are major contributors to viral hepatitis, with HCV significantly increasing the risk of developing hepatocellular carcinoma [2].

Understanding the interactions between the host’s immune responses and persistent viruses is essential for developing effective treatments and vaccines. The innate immune response constitutes a potent first line of defense involving various immune cells such as dendritic cells (DCs), macrophages, and natural killer (NK) cells. Interferons (IFNs), the central cytokines in this response, induce an antiviral state in host cells and regulate components of innate and adaptive immunity response [2,3,4]. Upon infection, host cells detect viral DNA or RNA using pattern recognition receptors (PRRs), including Retinoic acid-inducible gene-I (RIG-I)-like receptors (RLRs), Toll-like receptors (TLRs), nucleotide-binding oligomerization domain (NOD)-like receptors (RNA sensors), Cyclic GMP-AMP (cGAMP) synthase (cGAS), interferon gamma-inducible protein 16 (IFI16), Absent in melanoma 2 (AIM2), and Dead-box helicase 41 (DDX41) [2]. In contrast to the rapid activation of the innate immune system, the adaptive immune response usually presents a notable delay of about 6 to 8 weeks before becoming detectable. This response involves the expansion and maturation of specific T and B cell subsets that contribute to viral clearance through humoral antibody and T cell-mediated responses [3].

**Figure 1:**
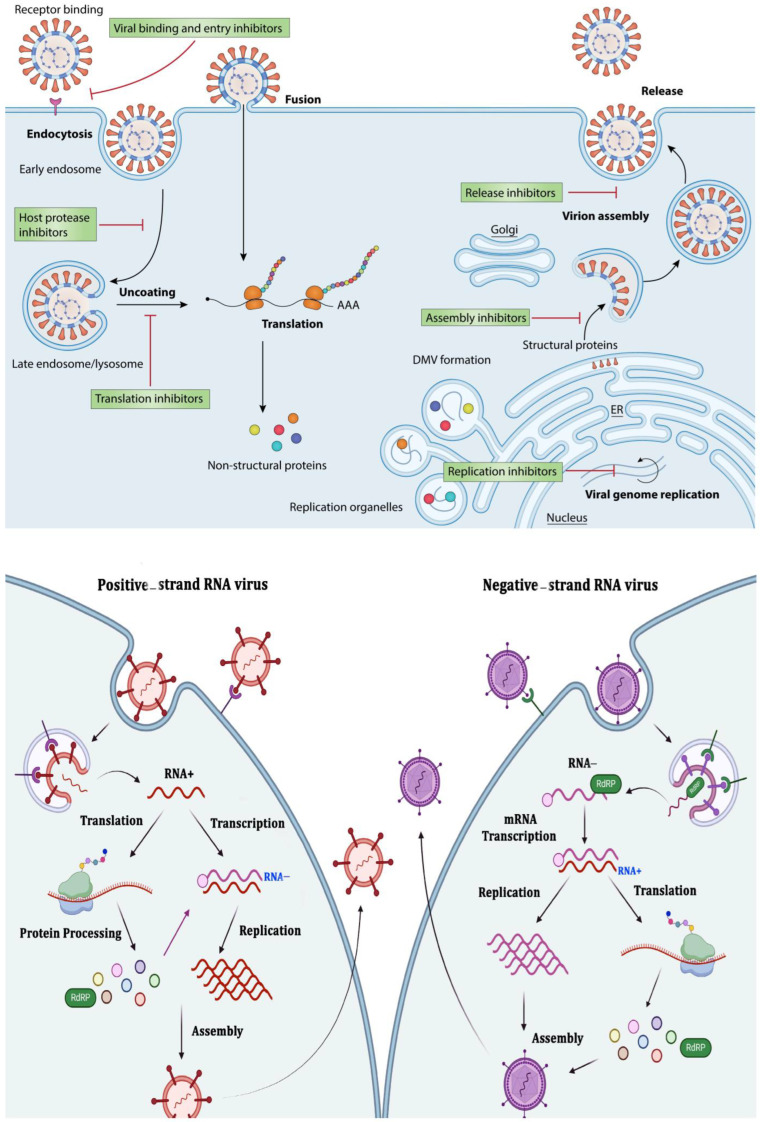
Viral replication cycle and the inhibitory targets. The replication cycle includes viral attachment, endocytosis, uncoating and translation, viral genome replication, virion assembly, and release. These steps can be targeted to inhibit the viral cycle.

B cells are involved in antibody-independent mechanisms during acute and chronic viral infections. Regulatory B cells (Bregs), which produce interleukin-10 (IL-10), are key in modulating immune responses and balancing beneficial and harmful effects on disease progression [5]. Bregs regulate T cell activity and enhance the immune response through cytokine secretion, antigen presentation, and Toll-like receptor (TLR) activation [6]. CD8+ T cells play a crucial role in eliminating intracellular pathogens. Their cytotoxic functions involve the release of cytolytic proteins such as granzyme B and perforin, along with cytokines like interferon-gamma (IFNγ) and tumor necrosis factor-alpha (TNFα). Strong CD8+ T cell responses are essential for controlling persistently replicating viruses, including lymphocytic choriomeningitis virus (LCMV), simian immunodeficiency virus (SIV), and hepatitis B virus (HBV) [7]. Additionally, robust CD8+ T cell activity is linked to protection in patients infected with HIV, HCV, and HBV. However, over time, these T cell responses can lead to viral escape mutations, and their functionality during chronic infections is often reduced compared to the potent effector and memory T cells generated during acute infections [2]. Hepatitis B and C viruses and HIV employ complex immune evasion strategies, including antigenic diversity, immune checkpoint modulation, and inhibition of antigen presentation. HBV produces subviral particles (SVPs) that act as decoys to evade immune recognition, whereas HCV disrupts interferon signaling pathways to suppress antiviral responses. HIV, through proteins such as Nef, downregulates MHC class I molecules and prevents effective cytotoxic T-cell responses. Understanding these mechanisms is critical for the development of targeted therapies that enhance immune clearance and neutralize viral persistence [8]. By examining host–virus interactions, this study aims to provide insight into novel therapeutic strategies that could reduce chronic viral infections and improve treatment outcomes; Figure 1.

The host factors that contribute to the viral entry, replication, and secretion of HBV, HCV, and HIV are shown in Table 1.

## 2. Molecular Pathways of Viral Evasion and Adaptation

Viruses have developed sophisticated strategies to evade host immune responses, contributing to the establishment of chronic infections. Key mechanisms include the following.

### 2.1. Antigenic Variation and Envelope Alteration

Many viruses, such as HIV and HBV, have high rates of mutation and recombination. This ability enables them to frequently change their surface proteins, including the envelope glycoprotein gp120 in HIV [9]. Additionally, these viruses can express an envelope complex that reduces access to antibodies. This capacity to modulate their antigenic profiles constitutes a fundamental strategy for evading the host immune response, complicating efforts to establish effective immunological defenses. In the context of HBV, sustained exposure to elevated levels of viral antigens, notably Hepatitis B surface antigen (HBsAg) and Hepatitis B e antigen (HBeAg), has been closely associated with the phenomenon of T cell exhaustion during chronic hepatitis B (CHB) infection [10,11]. This condition results from prolonged antigenic stimulation, leading to a progressive decline in T-cell functionality and an impaired immune response against the virus [12]. Intriguingly, subviral particles (SVPs) produced by HBV are composed exclusively of envelope proteins and are generated in quantities that far exceed those of infectious virions. The prevailing scientific consensus indicates that these SVPs play a pivotal role in immune evasion by serving as decoys for the immune system [13]. Thereby shielding the infectious Dane particles from neutralizing humoral responses. Notably, SVPs can also bind to hepatocytes, which may play a role in the HBV life cycle and immune interactions [14].

### 2.2. Viral Epigenetic Modifications

Viral RNA undergoes various epigenetic modifications, including N6-methyladenosine (m6A), 5-methylcytosine (m5C), and N4-acetylcytidine (ac4C), which are crucial for viral replication by regulating RNA splicing, stability, and translation [15]. Notably, m6A acts as a molecular signature that facilitates immune evasion, particularly in HBV. In HIV-1, m5C modification on viral mRNA is mediated by NSUN2; loss of NSUN2 disrupts alternative splicing and ribosomal recruitment, impairing viral replication. Evidence suggests that m5Cs and the host methyltransferase NSUN2 play crucial roles in the inhibition of interferon (IFN) by HBV [16].

### 2.3. Non-Neutralizing Antibodies

Non-neutralizing antibodies (nnAbs) play a complex role in the immune response to viral infections. Unlike neutralizing antibodies, which can directly inhibit viral entry into host cells, nnAbs bind to viral antigens without blocking infection. This duality can contribute to immune evasion; nnAbs can bind to viral proteins, such as gp120 in HIV, effectively obstructing the recognition of viral epitopes by neutralizing antibodies and cytotoxic T lymphocytes (CTLs) [17]. Non-neutralizing antibodies (nnAbs) play a multifaceted role in the immune response to chronic viral infections. Unlike neutralizing antibodies, which directly prevent viral entry into host cells, nnAbs bind to viral antigens without inhibiting infection. This interaction can aid immune evasion by hindering the recognition of viral epitopes by neutralizing antibodies and CTLs [18]. In the context of HIV infection, nnAbs target gp120, a key envelope glycoprotein, forming immune complexes that reduce the effectiveness of neutralization and promote viral persistence. Similarly, during HBV infection, subviral particles (SVPs) consisting solely of envelope proteins act as decoys, diverting the immune response away from infectious virions and shielding them from neutralizing antibody activity. Furthermore, nnAbs can contribute to antibody-dependent enhancement (ADE) of infection. In this process, virus–antibody complexes facilitate viral entry into Fc receptor-expressing cells, potentially exacerbating the infection. Understanding the intricate dynamics between nnAbs and immune evasion strategies sheds light on how chronic viruses manage to persist despite active immune surveillance [19].

Given the role of nnAbs in immune evasion and viral persistence, several therapeutic strategies are being explored to counteract their effects and enhance protective immune responses. One approach involves the development of next-generation vaccines aimed at preferentially inducing broadly neutralizing antibodies (bNAbs) [20]. For example, in HIV research, sequential immunization strategies are being used to guide B cell maturation toward the production of bNAbs capable of neutralizing a wide range of viral strains. Beyond vaccine innovations, targeting viral decoy mechanisms offers another promising avenue. Chronic viruses such as HBV and HCV often produce excessive non-infectious viral proteins or particles that divert immune responses away from neutralization; disrupting these mechanisms could enhance immune efficiency [21]. Additionally, monoclonal antibodies designed to block Fc-mediated uptake of nnAb-virus complexes by immune cells are being investigated to reduce antibody-dependent enhancement (ADE) and limit immune evasion. Another exciting strategy involves combining immune checkpoint inhibitors such as anti-PD-1 and anti-CTLA-4 with bNAb therapy. Preclinical studies suggest this combination rejuvenates exhausted T cells, enabling them to more effectively clear infected cells, even in the presence of nnAb-driven challenges. Furthermore, therapeutic vaccines are being designed to stimulate stronger CD8+ T cell responses to compensate for inadequate antibody-mediated immunity. T cell receptor (TCR)-based immunotherapies are also under investigation to bypass nnAb-related immune evasion altogether. By counteracting the immunosuppressive effects and evasion strategies linked to nnAbs, these therapeutic interventions hold great promise for improving immune control over chronic viral infections [22].

### 2.4. Host Cell Tropism and Tissue Reservoirs

Viruses employ several mechanisms to evade the immune response, one of which involves the inhibition of apoptosis through the production of caspase inhibitors and specific tropism factors [23]. Viral tropism denotes a virus’s inclination to infect particular cells or tissues within a host organism. This preference is predominantly determined by the presence of specific membrane receptors on the surface of host cells, which interact with viral proteins referred to as “antireceptors.” The interaction between these receptors and viral proteins enables the virus to penetrate the host cell’s cytoplasm, resulting in infection. For instance, in the case of human immunodeficiency virus (HIV), the hypervariable region 3 (V3) of the viral envelope glycoprotein gp120 is instrumental in influencing tropism by facilitating the virus’s association with cellular chemokine receptors, specifically CCR5 and CXCR4. Likewise, for the HBV, its tropism is directed toward hepatocytes in the liver, as it binds to specific sodium taurocholate cotransporting polypeptide receptors. This binding process enables the virus to enter liver cells and subsequently replicate within them [24].

### 2.5. Latency and Integration

**Figure 2:**
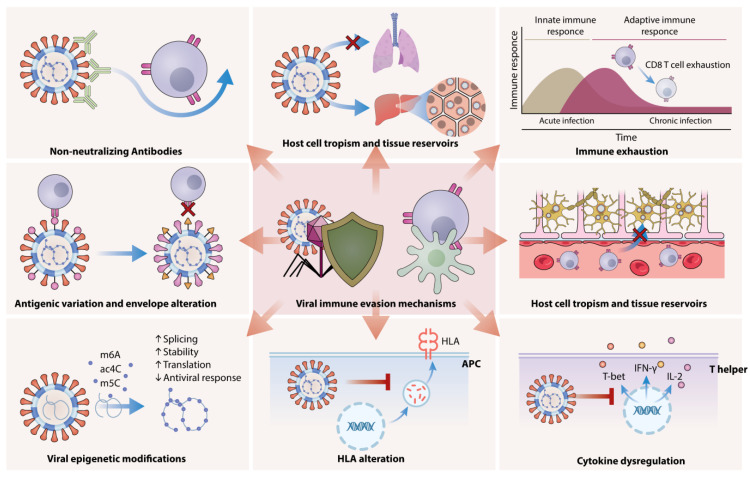
A summary of different viral immune evasion mechanisms.

The primary strategies employed by viruses during chronic infections include continuous replication, latency with the potential for reactivation, and integration into the host genome. Latency is a reversible state of nonproductive viral infection within host cells, which allows viruses to evade the immune system and remain undetected. Viral latency is a complex process that is not yet fully understood. It is influenced by various viral factors, the characteristics of the infected cell type (such as neurons or myeloid cells), and both innate and adaptive host responses [25]. During this phase, viruses can infect nonpermissive or semipermissive host cells and persist in “immune privileged” tissues such as the brain, retina, and kidneys. For instance, Herpes Viruses (HSV-1, HSV-2, VZV, CMV, EBV) establish latency in specific cell types (neurons, myeloid cells, B cells) and can reactivate and cause CNS inflammation and ocular immune disorders [25]. CMV establishes latency in hematopoietic cells, rendering them inactive or anergic, which in turn promotes further latency. Human cytomegalovirus (HCMV) produces microRNAs that regulate various processes in infected cells, contributing to HCMV pathogenicity and viral latency [26]. HIV-1 remains in a transcriptionally inactive form as a provirus within the genome of CD4+ T cell memory cells. These memory cells lack the necessary transcription factors for viral replication. This capability to integrate into the host genome and establish latency complicates treatment efforts and leads to chronic infection. Additionally, macrophages, like CD4+ T cells, are also susceptible to HIV infection and play a significant role in viral latency and cell-to-cell transmission, even in individuals undergoing antiretroviral therapy [24]; Figure 2.

## 3. Viral Evasion of Host Immunity Through HLA Alteration, Immune Exhaustion, and Cytokine Dysregulation

Viruses can directly suppress the host’s antiviral immune response by altering the regulated expression of MHC class molecules on antigen-presenting cells (APCs). This disruption of normal HLA presentation on APCs plays a critical role in impairing the initiation of an effective immune response against viral infections. Moreover, chronic viral infections are often associated with immune exhaustion, where T cell functionality and cytokine production become dysregulated. Additionally, several viruses have developed mechanisms to bypass JAK/STAT signaling, and irregular JAK/STAT signaling is associated with immune dysregulation [24]. Moreover, viruses have several distinct strategies to prevent the death of infected host cells, including the production of caspase inhibitors (proteases that cleave cellular proteins), homologs of Bcl-2 (apoptosis inhibitory proteins), and FLICE-like inhibitory proteins (FLIPs) [24]. 

IFNs regulate a broad spectrum of genes, primarily through the activation of interferon-stimulated genes (ISGs), which influence apoptotic pathways. Dysregulated IFN signaling can alter the expression of anti-apoptotic proteins like BCL2 and FLIP. Type I and II IFNs, notably IFN-α, IFN-β, and IFN-γ, can either facilitate or suppress apoptosis depending on the cellular context and associated signaling mechanisms [27]. For instance, IFN-γ is known to reduce BCL2 expression in certain immune and cancer cells, thereby promoting apoptosis through STAT1-dependent pathways. Conversely, IFNs can also enhance the expression of pro-apoptotic BCL2 family members such as BAX and PUMA, shifting the balance towards apoptosis. IFNs, particularly IFN-γ, are also capable of suppressing FLIP expression, increasing cell sensitivity to apoptosis via death receptors like Fas and TRAIL-R [28]. This process is often exploited in immune responses against infected or malignant cells. However, some viruses can manipulate IFN pathways to maintain FLIP expression, thereby evading immune-induced cell death. Consequently, dysregulated IFN signaling, whether excessive or insufficient, can lead to aberrant BCL2 and FLIP expression, impacting cell survival, immune evasion, and disease progression [29].

HIV employs various strategies to downregulate MHC class I and II expression; similarly, CMV downregulates MHC class I molecules on infected cells, decreasing their visibility to CTLs [30]. This suppression is primarily mediated by the HIV-1 accessory protein Nef, which interferes with the transcription and translation of MHC genes, resulting in reduced surface expression on infected cells. HBV infection reduces the expression of HLA-DP and HLA-DQ molecules on APCs. This decrease weakens their ability to present antigens, leading to an inefficient T-cell response. Additionally, polymorphisms in HLA-II genes during HBV infection can alter the antigen-binding properties of HLA-II, which may affect the HBV-specific immune response and contribute to the persistence of the HBV infection [12,31].

T-cell exhaustion was first identified in mouse models of chronic lymphocytic choriomeningitis virus (LCMV) infection. In these models, antigen-specific CD8 T cells progressively lost their effector functions and exhibited a diminished ability to kill virally infected cells. While an activated T-cell response can usually cause significant damage to targeted cells and effectively control and eliminate foreign invaders, conditions of chronic infection create a different scenario [32]. Prolonged exposure of T cells to high levels of antigen leads to a severe dysfunctional state known as T-cell exhaustion. Several transcription factors have been associated with exhausted T cells, including NFAT, Batf, IRF-4, T-bet, Eomes, and Blimp-1 [33]. However, no master regulator of T-cell exhaustion has been identified. CD8+ T cells show reduced secretion of key cytokines such as IL-2, IFN-γ, and TNF-α, along with diminished cytotoxic and proliferative abilities. Exhausted T cells also have altered metabolism and poor memory recall abilities. These cells typically express multiple inhibitory receptors like PD-1, which resemble dysfunctional T-cell phenotypes. Recent research indicates that exhausted CD8+ T cells have elevated levels of transcription factors such as IL- 4, basic leucine zipper ATF-like transcription factor, and nuclear factor of activated T cells 1. These factors promote the expression of various inhibitory receptors, including PD-1, and are associated with impaired antiviral function and cellular metabolism. T-cell exhaustion occurs in human chronic viral infections, including HIV, HCV, HBV, and HTLV-1, as well as cancer [12,32].

In the context of HIV, the virus increases the expression of inhibitory receptors such as PD-1 and CTLA-4, which impair T-cell function. At the same time, HIV downregulates the expression of key transcription factors and cytokines essential for effective T-cell responses, including the T-box transcription factor (T-bet), IFN-γ, and IL-2. As a result, exhausted CD4 T cells show reduced capacity to proliferate in response to HIV and exhibit a diminished polyfunctional cytokine response, primarily due to decreased production of IL-2. Additionally, cytokines like IL-10 are also implicated in the phenomenon of T-cell exhaustion during HIV infection [32]. HIV can kill infected T helper cells by inducing apoptosis and increasing levels of Fas, FasL, and TNFα while also reducing Bcl-2 expression and activating p53. This loss weakens the immune system’s ability to produce vital cytokines, allowing the virus to persist and replicate. Additionally, the HIV proteins Vpu and Nef disrupt JAK/STAT activation by IFN-α, diminishing its effectiveness while promoting the degradation of Type 1 IFN and suppressing interferon-stimulated genes. In the case of chronic HBV infection, exhausted HBV-specific CD8+ T cells also express multiple inhibitory receptors, including PD-1, CTLA-4, 2B4, Tim-3, and lymphocyte activation gene 3. This expression closely resembles the transcriptional profiles of CD8+ T cells. Additionally, the decreased secretion of pro-inflammatory cytokines, such as IL-2, IFN-γ, and IL-21, by HBV-specific CD4+ T cells contributes to the exhaustion of CD8+ T cell responses during chronic HBV infection. Persistent antigen exposure leads to chronic T-cell activation, eventually resulting in exhaustion [12].

Altered cytokine production can significantly impact the immune landscape, often leading to a pro-inflammatory environment that contributes to tissue damage and sustains chronic inflammation. CMV primarily suppresses and modulates innate immune responses by inhibiting IFN-I production through its envelope glycoproteins. HBV upregulates PD-L1 and induces DCs that exhibit extremely low T cell-stimulatory capacity and produce IL-12. Consequently, HBV infection can lead to deficient secretion of inflammatory cytokines such as IL-12 and IFN-α/β in response to pathogen-associated molecular patterns (PAMP) stimulation, further dampening the third signal required for CD8+ T cell activation. CD4+ T cells can also differentiate into CD4+ CD25+ Foxp3+ regulatory T cells (Tregs), which secrete the suppressive cytokines IL-10 and transforming growth factor-beta (TGF-β), resulting in a progressive loss of HBV-specific CD8+ T cells [34]. Moreover, HBV-related antigens and HBV-derived exosomes dampen the signaling pathways of Retinoic acid-inducible gene I (RIG-I), nuclear factor kappa B (NF-kB), and p38 mitogen-activated protein kinase, leading to the functional suppression of NK cells during chronic hepatitis B infection. Dysfunction of T follicular helper (Tfh) cells may impair humoral immune responses during chronic hepatitis B (CHB) infection. Additionally, HBcAg binding to B cells induces other inhibitory receptors like Fc receptor-like 4 (FcRL4) and FcRL5, resulting in dysfunctional B cell responses. HBcAg also promotes the differentiation of regulatory B cells (Breg cells) that produce IL-10. These Breg cells help suppress CD8+ T cell responses and support the formation of regulatory T cells, ultimately facilitating immune tolerance. Although B cells function as professional APCs, their reduced expression of co-stimulatory molecules such as CD80 and CD40 may impair interactions with T cells, leading to T cell exhaustion [12].

## 4. Host Genetic Factors Influencing Infection Outcomes

Understanding the genetic factors that affect outcomes in chronic viral infections is essential for creating personalized treatment strategies. Insights from genome-wide association studies (GWAS) can help identify individuals at higher risk and guide the development of targeted vaccines and interventions. More research, particularly in underrepresented populations, will enhance our knowledge of the genetics involved, leading to better health outcomes. However, environmental factors, underlying diseases, and co-infections and their impact on disease severity and clinical outcomes should not be ignored [35,36].

### 4.1. Human Leukocyte Antigen (HLA) Diversity

The HLA genes play a crucial role in the immune response to viral infections. Strong signals indicating an immune response to various viral antigens have been detected in the HLA region, with a notable prevalence of associations linked to alleles and amino acids in HLA-DRB1 and HLA-DQB1. Additionally, there are transcriptome-level associations involving multiple class II and III HLA genes [35]. The influence of HLA polymorphisms on chronic viral infections, particularly HIV, plays a pivotal role in shaping disease progression and immune control. Variants like HLA-B*57 and HLA-B*27 are strongly linked to slower HIV progression due to their superior capacity to present viral peptides to cytotoxic T cells, promoting more robust immune responses [37]. Conversely, certain HLA variants, including HLA-B*35-Px and HLA-C*07, are associated with faster disease progression as they are associated with suboptimal immune responses. Furthermore, regulatory single nucleotide polymorphisms (SNPs) within the MHC region, such as rs9264942 near HLA-C, influence HLA expression levels and contribute to varying degrees of HIV viremia control. These genetic factors interact with broader viral immune evasion strategies, notably HIV’s ability to downregulate MHC class I molecules through accessory proteins like Nef, thereby weakening cytotoxic T-cell recognition [38]. Specific alleles within the HLA system can significantly influence the ability to control and eliminate chronic infections. For example, certain HLA-DP alleles have been associated with the persistence of HBV, underscoring their role in modulating T-cell responses. Research indicates that individuals carrying specific HLA-DPB1 variants are more susceptible to chronic HBV infection, suggesting a genetic predisposition that affects the immune system’s recognition of the virus [36]

### 4.2. Immune Regulators

Genetic variations in immune regulatory genes such as CD40 and INTS10 have been identified as potential influencers on the efficacy of the immune response to chronic viral infections. These genes are involved in signaling pathways critical for T cell activation and differentiation, which can alter the host’s ability to respond effectively to HBV or HIV [36].

### 4.3. Ethnic and Genetic Variation

Ethnic and genetic variations play a significant role in determining susceptibility to and outcomes of infectious diseases, particularly viral infections such as HBV and HCV. For example, the HLA DR13 allele provides a level of protection against vertical transmission of both HBV and HCV in neonates from Chinese and Italian populations [39]. However, various alleles are correlated with differing levels of susceptibility within these groups. Additionally, polymorphisms in host genes can alter gene function, leading to either protective or harmful effects against diseases. Several common host genes and their polymorphisms are involved in the response to infectious diseases [40].

## 5. Targeting Host–Virus Interactions for Chronic Infection Therapies

The ongoing threat posed by viral infections and the growing need for effective treatments has led to a significant transformation in the field of antiviral strategies in recent years [41]. Although vaccines are frequently the most effective strategy for long-term control of viral infections, their effectiveness is somewhat constrained by the significant time and effort required for widespread administration, as well as their inability to deliver complete antiviral protection. Consequently, it is essential to develop antiviral treatments to address emerging viruses or infections when preventive measures are insufficient [42]. Currently, the majority of FDA-approved antiviral drugs are designed to target viral proteins critical to the replication cycle. However, a significant challenge with these antiviral agents lies in the inherent propensity of viral mutations during replication, which allows many viruses to adapt to these interventions and develop resistance [43]. Host-directed therapies (HDTs) offer a novel and promising strategy for addressing viral infections by targeting host proteins essential for viral replication or by modulating the host’s immune response [44].

In contrast to conventional antiviral approaches, HDTs overcome the issue of resistance by concentrating on host factors, which are commonly exploited by diverse viruses through shared cellular pathways. Recent advancements in genomics and proteomics have facilitated the identification of new host targets, paving the way for the development of broad-spectrum treatments that both suppress viral replication and improve clinical outcomes [45]. A key challenge in the discovery and development of HDTs lies in the necessity for a comprehensive understanding of virus–host interactions and their biological relevance to viral replication. Specifically, only those host factors that play a critical role in supporting viral replication while being amenable to modulation without disrupting essential endogenous molecular functions are viable candidates for pharmacological inhibition in HDT approaches [46]. Recent advances have introduced innovative experimental approaches for identifying cellular host factors, offering robust tools to investigate both direct and indirect host–pathogen interactions [46]. Furthermore, it is possible to alter the course of an infection by manipulating some host responses to a viral infection, such as the interferon (IFN) and adaptive immune responses. Over the past two decades, host-directed therapies have gained considerable attention. However, only a small number of these studies have FDA-approved drugs, while the majority are still in the preclinical stages [45].

## 6. Identifying Key Host Targets for Therapeutic Intervention

The lengthy process of drug development frequently restricts its utility in addressing emerging viral outbreaks, underscoring the necessity for strategies that facilitate the swift identification of antiviral agents [42]. Only a small number of the various methods that have been successfully used to identify host dependency factors (HDFs) have been successfully converted into efficient HDT to date. The use of cyclosporine for influenza A virus (IAV), C-C chemokine receptor type 5 (CCR5) antagonists for HIV, and antioccludin and anti-claudin-1 monoclonal antibodies for hepatitis C virus (HCV) are examples of effective HDT [43,46]. The use of high-throughput screening methods to identify novel host targets essential for viral replication has been made feasible by recent advancements in proteomics and genomics [44]. In general, modern technologies can be categorized based on the type of technique employed, encompassing functional genomics approaches, genomic-based strategies, proteomic-based strategies.

The host factors identified as vital in supporting the life cycles of HBV, HCV, and HIV play key roles in facilitating viral entry, replication, immune evasion, and persistence. HBV predominantly relies on the sodium taurocholate cotransporting polypeptide (NTCP) receptor to enter hepatocytes, whereas HCV utilizes a combination of receptors, including CD81, occludin, and claudin-1. In contrast, HIV targets CD4+ T cells by binding to the CD4 receptor and utilizing CCR5 or CXCR4 as coreceptors. During viral replication, HBV exploits epigenetic modifications such as N6-methyladenosine (m6A) to enhance RNA stability and translation. HCV utilizes host microRNA-122 to boost genome replication, while HIV employs host transcription factors like NF-κB and Sp1 to facilitate its replication and integration into the host genome [47]. Host factors are also integral to viral immune evasion mechanisms. HBV subverts immune responses by downregulating HLA-DP and HLA-DQ expression, thereby disrupting antigen presentation. HCV evades detection by suppressing interferon-stimulated genes (ISGs), and HIV utilizes its Nef protein to downregulate MHC class I molecules, preventing cytotoxic T-cell recognition and clearance. Gaining a deeper understanding of these host–virus interactions is crucial for the development of host-directed therapies (HDTs). These therapies aim to target host dependency factors instead of viral proteins, presenting a promising approach to counteract drug resistance and enhance treatment outcomes [12].

### 6.1. Functional Genomics Approaches

Our knowledge of how human pathogenic viruses use our cells to replicate has significantly increased due to functional genomics. RNA interference (RNAi), haploid cell screening, and the innovative gene editing of the CRISPR/Cas9 system are key technologies in this approach.

### 6.2. RNA Interference

RNA interference (RNAi) is a cellular mechanism that modulates gene expression by processing double-stranded RNA (dsRNA) into small RNA molecules, including small interfering RNA (siRNA) and microRNA (miRNA) [48]. These small RNAs can selectively bind to messenger RNA (mRNA), resulting in its degradation or the suppression of its translation. Host genes that facilitate viral replication can be found using this approach. While siRNA-based screens have revealed numerous cellular components, such as host cellular receptors, transcription factors, kinases, and transporter proteins, as critical HDFs for viral replication, RNAi screenings face several limitations. These include variations in gene silencing efficiency across different siRNAs, limited consistency in the overlap of top candidate genes, and the potential for triggering innate immune responses [46].

In spite of its drawbacks, RNAi screens have effectively yielded useful information, including druggable targets for newly developing viruses, such as the ubiquitin ligase CBLL1 implicated in the internalization of the West Nile Virus (WNV) [49]. Another study utilizing a genome-wide siRNA screen identified the IκB kinase (IKK)-NF-κB signaling pathway as a critical host mechanism required for the viral life cycle. Targeted inhibition of this pathway markedly suppressed viral replication, highlighting its potential as a promising druggable target for therapeutic development. Furthermore, researchers developed an innovative siRNA-peptide dendrimer delivery system designed to target SARS-CoV-2 viral genes essential for replication. The study demonstrated that the modified siRNA effectively suppressed viral gene expression, leading to a significant reduction in viral replication within infected cells [50].

### 6.3. Haploid Cell Screening

Haploid screening, a technique that utilizes cells with a single allele per gene, enables the efficient generation of null mutants for most nonessential genes. This method has proven to be an invaluable tool for identifying numerous HDFs, which are useful for understanding host–pathogen interactions [51]. A study highlights Niemann-Pick C1 (NPC1) as an essential host factor for Ebola virus (EBOV) entry and replication. Using haploid screening, researchers demonstrated that NPC1 facilitates viral fusion with host cell membranes, a critical step for releasing viral genetic material. Mutations in NPC1 significantly inhibited EBOV infection, underscoring its potential as a therapeutic target for antiviral interventions [52]. Moreover, another study that used a two-color haploid genetic screening method more recently found 69 new candidate genes linked to HIV latency maintenance. Several druggable host factors were identified among them, including ADK, NF1, and GRIK5 [53].

### 6.4. CRISPR Screening Technologies

An adaptive immune system made up of prokaryotic Clustered Regularly Interspaced Short Palindromic Repeats/CRISPR-CRISPR Associated Proteins (CRISPR/Cas) defend bacteria against bacteriophages and plasmids. The modularity and ease-of-use nature of CRISPR/Cas has facilitated a broad range of applications, particularly in virology. In this field, CRISPR/Cas is employed to investigate HDFs through loss-of-function and gain-of-function studies, thereby enhancing our understanding of host–virus interactions [54]. CRISPR-based genetic screens have significantly advanced our understanding of host-dependent factors crucial for the life cycles of viral pathogens, including Zika virus, dengue virus, and influenza virus. These studies have also uncovered novel host factors, such as WDR7, CCDC115, CYTH2, and TMEM199, which are crucial for the replication and pathogenesis of the influenza virus. Furthermore, using this screening approach, novel host factors involved in SARS-CoV-2 infection have been identified, including key components of the TGF-β signaling pathway and the chromatin remodeling complex. These pathways are critical for supporting viral replication, as demonstrated by a significant reduction in viral load when pathway inhibitors are applied. Similarly, recent CRISPR screening studies have identified the GATA6 gene as an HDF that regulates the expression of ACE2, a key receptor facilitating SARS-CoV-2 entry into host cells [55].

### 6.5. Genomics-Based Strategies

Exploring the impact of human genetics on susceptibility to infectious diseases provides a valuable opportunity to uncover novel insights into the mechanisms of host–pathogen interactions. Such understanding not only enhances our knowledge of disease biology but also aids in identifying potential drug targets and therapeutic strategies [56]. Genomic-based methodologies are broadly categorized into Genome-Wide Association Studies (GWAS), RNA-Sequencing-based Expression Profiling, and Single-Cell RNA-Sequencing (scRNA-Seq). Advancements in genetic technologies have facilitated the discovery of numerous genetic loci associated with susceptibility to infectious diseases through GWAS. These studies have identified key genetic factors that influence the risk and progression of diseases such as HIV, hepatitis, tuberculosis, and malaria [57]. GWAS serves as an invaluable methodology for identifying novel HDFs by analyzing hundreds of thousands of genetic variants across the genomes of large populations. This approach seeks to link specific allelic variations with particular phenotypic traits or disease susceptibilities. A primary focus of GWAS is the examination of SNPs, which represent common sequence variations in the human genome. SNPs associated with the interferon lambda gene, IFNL3 or IFNL4, have been shown to have a significant correlation with the efficacy of treatment using pegylated interferon-alpha and ribavirin (PEG-IFN/RBV) for HCV infection [58].

A severe COVID-19 GWAS Group has identified genetic variants linked to genes such as SLC6A20, LZTFL1, CCR9, FYCO1, CXCR6, and XCR1 in patients experiencing respiratory failure due to COVID-19. Similarly, in patients with COVID-19 pneumonia, GWAS have been useful in uncovering genetic deficiencies in the TLR3/TLR7-dependent type I interferon response. These findings highlight critical genetic factors that may influence disease severity and immune responses in affected patients [59]. The advancement of NGS technology has made it possible to globally profile mRNA changes in response to viral infections. RNA sequencing (RNA-seq) enables the identification of genes with statistically significant changes in expression, whether upregulated or downregulated, using computational differential expression analysis. This process often integrates gene expression profiles with experimental metadata, such as virus type, cell origin, or time points. Additionally, databases are utilized to assign functions to previously uncharacterized genes and to elucidate the regulatory pathways disrupted by the infection [46].

A study employed comparative transcriptome analysis through RNA sequencing to investigate host cell responses to SARS-CoV-2 infection, uncovering significant changes in gene expression. Among the findings, the study emphasized the efficient entry of SARS-CoV-2 into host cells, facilitated by the upregulation of the TMPRSS2 gene, which was identified as a critical factor contributing to the virus’s high infectivity [60]. Single-cell RNA sequencing (scRNA-Seq) addresses the limitations of bulk studies by analyzing individual cells, offering detailed insights into host–virus interactions. It identifies susceptible cell types, characterizes novel immune subtypes, examines gene expression changes like pro-inflammatory responses, and maps cellular interaction networks affected by viral infections [61].

### 6.6. Proteomics-Based Strategies

Proteomics techniques enable the detailed analysis of protein–protein interactions and infection-induced signaling alterations, facilitating the study of structure–function relationships. Understanding these interactions between viral and host proteins is crucial for the development of effective treatments and preventive strategies against infectious diseases. Key proteomic approaches include mass spectrometry, yeast two-hybrid screening, and imaging-based techniques, each offering valuable insights into the molecular dynamics of host–pathogen interactions [62]. Mass Spectrometry (MS) is an advanced technique used to identify and analyze host proteins that interact with viral components during various stages of the viral life cycle, such as replication, entry, and immune evasion. These host proteins can serve as potential therapeutic targets for antiviral drug development, offering new opportunities for disrupting viral replication and preventing infection. The spread of contagious pathogens such as SARS-CoV-2 and HIV underscores the urgent need for cutting-edge diagnostics and therapeutic approaches. Biomaterials based on aggregation-induced emission (AIE), known for their exceptional biocompatibility and fluorescent capabilities, present promising solutions for detecting and preventing these diseases. Progress in proteomics and bioimaging has further amplified the potential of AIE-based technologies in addressing the challenges posed by emerging infectious diseases [63,64].

The Yeast Two-Hybrid (Y2H) system is a commonly employed molecular biology tool for investigating protein–protein interactions in a high-throughput context. This technique is especially valuable for identifying novel host factors implicated in virus–host interactions by detecting direct binding events between viral and host proteins. The method relies on the reconstitution of a split protein, where fragments of the protein are rejoined to restore its functional activity, enabling the detection of interactions [65]. The Y2H approach has facilitated the identification of several HDFs that interact with HIV-1 integrase, a critical enzyme for viral integration. These include key factors such as LEDGF/p75, Transportin-SR2 (TNPO3), von Hippel–Lindau binding protein 1 (VBP1), and SNF5, all of which play essential roles in the integration of the viral genome into the host cell’s DNA. Moreover, the Y2H screening method identified key interactions between viral proteins Vpx (SIV/HIV-2) and Vpr (HIV-1) and the host protein Cyclin L2. These interactions regulate viral replication in quiescent cells and macrophages by modulating the host restriction factor SAMHD1. Cyclin L2 promotes SAMHD1 degradation in a DCAF1-dependent manner, enabling efficient viral replication in these normally restrictive environments. This highlights Cyclin L2 as a crucial HDF for HIV replication in non-cycling cells. In order to better understand viral structures and the interactions between host proteins and viral counterparts within the cell, advanced imaging methodologies such as high-resolution light microscopy, transmission electron microscopy (TEM), scanning electron microscopy (SEM), and confocal microscopy, use new optical imaging tools to perform spatiotemporal evaluations of the viral replication cycle [66].

### 6.7. Strategies for Modulating Host Immune Responses

Chronic viral infections, such as those caused by HIV, hepatitis B (HBV), or hepatitis C (HCV), pose significant challenges due to the virus’s ability to evade and suppress host immunity [67]. Modulating the host immune response offers a promising strategy to combat these infections by either strengthening the immune system or restoring its normal functionality. In addition to the previously established approaches, innovative therapeutic strategies targeting specific components of the immune response are being actively developed and tested for various viral infections. These advancements aim to facilitate the creation of broad-spectrum antiviral agents effective against multiple viruses or highly specialized treatments tailored to a particular virus, thereby minimizing complications and improving clinical outcomes. Immune regulation therapies present promising approaches for addressing M1-driven viral myocarditis. To improve targeting efficiency, researchers have designed engineered M2-derived extracellular vesicles incorporating cardiac-targeting peptides and platelet membranes [68].

### 6.8. Cytokine-Based Therapies

Cytokines are vital small proteins that facilitate communication within the immune system. They are key regulators of immune functions, hematopoiesis, and inflammation. Cytokine-based therapies, such as interferons (IFNs), interleukins (ILs), tumor necrosis factors (TNFs), and colony-stimulating factors (CSFs), are effective tools for managing viral infections [69]. Interferon-based treatments have shown great potential in viral infections. In order to treat chronic infectious diseases like hepatitis B and C, interferon-alpha (IFN-α) has been utilized extensively. According to preliminary clinical trials, IFN-β may control inflammatory responses and decrease viral replication, offering a possible treatment option for SARS-CoV-2 infections. Moreover, a study examines the roles of interleukins IL-7 and IL-15 in enhancing immune responses during HIV-1 infection. IL-7 supports T-cell proliferation, while IL-15 enhances NK and CD8+ T-cell activity. These cytokines help sustain immune functionality, offering potential in cure strategies, including HIV-1 remission approaches [70,71].

### 6.9. Checkpoint Inhibition Therapy

Checkpoint inhibition treatment involves the use of monoclonal antibodies (mAbs) to block the interactions between immunosuppressive receptors and their corresponding ligands. This approach primarily targets key proteins such as programmed cell death 1 ligand 1 (PD-L1), cytotoxic T-lymphocyte-associated protein 4 (CTLA-4), and mucin-domain-containing protein 3 (TIM-3). By inhibiting these pathways, checkpoint inhibitors enhance the activation of effector T-cells, thereby promoting a more robust immune response. This strategy holds significant potential for improving immune responses against various diseases, including cancer and viral infections. Cao et al. demonstrated that inhibiting the CTLA-4 checkpoint molecule enhances the activity of hepatitis B virus (HBV)-specific CD8+ T cells both in the peripheral blood and within the liver, showing a potential strategy for boosting the immune response in chronic hepatitis B patients [72]. Moreover, inhibition of PD-1 has been shown to enhance the immune system’s responsiveness by promoting the production of interferon-gamma (IFN-γ) in HIV- and HBV-specific cytotoxic T cells. Using immune checkpoint inhibitors, such as anti-PD-1 antibodies, HDTs has shown encouraging results in virally associated cancers, especially those involving tumors linked to the human papillomavirus (HPV). These inhibitors work by enhancing the host’s immune system to recognize and attack cancer cells that have been infected by the virus [73].

### 6.10. Adoptive Cell Therapy

Adoptive Cell Therapy (ACT) has emerged as a highly promising approach for treating chronic viral infections such as HIV, hepatitis B (HBV), and hepatitis C (HCV) [74]. This innovative strategy involves the expansion and reinfusion of immune cells, which may be sourced either from the patient (autologous) or from a donor (allogeneic). Through genetic modifications or other methods of enhancement, ACT aims to optimize the functional capacity of these immune cells, enabling them to more effectively recognize and combat viral infections. Based on around twenty completed phase I/II clinical trials and more than thirty continuing clinical trials, transfusion of virus-specific T cells (VSTs) is an effective treatment for viral infections. Genetic engineering of VSTs using chimeric antigen receptors (CARs) is an emerging strategy aimed at enhancing their longevity and functional efficacy. Current research is applying this approach to address infections caused by HIV, HBV, HCV, and coronaviruses [75].

Targeting viral proteins, primarily gp-120, with CD4 and/or CD8 CAR T cells in clinical and preclinical trials (NCT03240328 and NCT04648046) showed significant suppression of HIV replication and destruction of HIV-infected cells; however, complete eradication of HIV-infected cells remains elusive. This limitation is attributed to the low expression of HIV antigens on the surface of infected cell membranes and insufficient CAR T-cell infiltration into HIV reservoirs. These challenges highlight the need for further optimization of CAR T-cell therapies to enhance their targeting precision and functional efficacy against HIV [76].

### 6.11. Therapeutic Vaccines

Development and evaluation of therapeutic vaccinations that target viral antigens may be a part of HDTs. Effective antiviral defenses are produced when the human immune system is strengthened against the virus by these adjuvant-based vaccinations. Vaccines require a comprehensive understanding of viral antigens and host immune responses, which can take time to develop. Nonetheless, they are crucial for preventing viral infections. Additionally, pandemics and newly emerging viral diseases present challenges that demand swift and adaptable solutions [77]. Adjuvants are used in vaccine manufacture to increase the efficacy of vaccines, strengthen immune responses, and promote protection against bacterial and viral infections. The evolution of adjuvants has revolutionized vaccine development by significantly improving immune protection against infectious diseases. These innovations have enabled the creation of vaccines that induce strong and durable immune responses. Toll-like receptor (TLR) agonists, such as monophosphoryl lipid A (MPL), represent a notable class of adjuvants. By stimulating innate immune pathways, they enhance the body’s defense mechanisms against viral antigens. For instance, MPL has been successfully integrated into vaccines for hepatitis B and SARS-CoV-2, where it has demonstrated the ability to substantially boost both antibody production and T-cell responses, thus enhancing overall vaccine efficacy [78].

**Figure 3:**
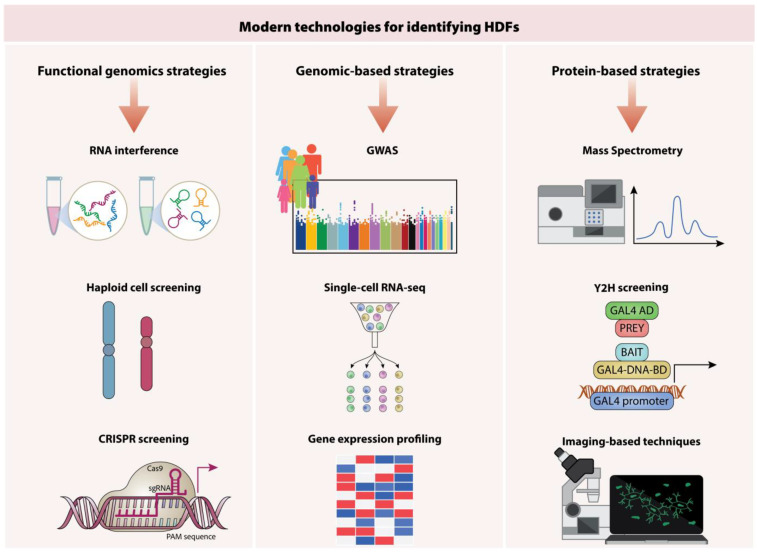
A summary of modern technologies for identifying viral host-dependence factors and the development of host-based therapeutic interventions. The use of modern technologies in the fields of functional genomics, genomics, and proteomics can significantly enhance our predictive capabilities regarding host–pathogen interactions and ultimately shape the future of host-based therapeutic interventions.

Likewise, TLR7/8 agonists, like imiquimod, are attractive adjuvants in influenza and HIV vaccines because they elicit a strong cellular immunological response, which makes them effective in generating a potent and durable immune response. Furthermore, a subunit vaccine featuring the stabilized prefusion SARS-CoV-2 spike protein combined with a CpG adjuvant has demonstrated strong immunogenicity in preclinical studies. This design significantly enhances antibody production and T-cell responses, with the CpG adjuvant, a TLR9 agonist, effectively activating innate immune pathways to boost the vaccine’s overall efficacy. The vaccine’s robust neutralizing activity against SARS-CoV-2 highlights its potential as a promising candidate for COVID-19 prevention [79]; Figure 3.

## 7. Role of Host–Virus Interactions in Therapeutic Innovations Potential of Immunotherapy in Chronic Viral Diseases

### 7.1. Antiviral Host Factors in Some Viral Diseases

The invasion of host cells by viruses, such as those that cause influenza, stimulates the host to release type I interferon (IFN). The detection of a viral infection occurs through pattern recognition receptors (PRRs), which are capable of identifying conserved viral components known as pathogen-associated molecular patterns (PAMPs). Various pathways may be triggered, resulting in the release of type I interferon based on the specific virus and the infected host cell. RNA viruses, including those responsible for influenza, are primarily identified by two types of pattern recognition receptors: Toll-like receptors (TLRs) and retinoic acid-inducible gene (RIG)-I-like receptors (RLR). Within endosomal areas, TLR3 and TLR7 are present and are responsible for recognizing double-stranded and single-stranded RNA, respectively, which triggers the expression of interferon [80,81]. RLR family proteins, notably RIG-I and melanoma differentiation-associated gene (MDA)-5, function within the cytoplasm of cells to recognize and bind to viral RNA. RIG-I detects short RNA with 5′-triphosphate caps produced during viral replication, while MDA-5 identifies longer viral RNA sequences and their replication stages. In the context of influenza A virus (IAV) infection, airway epithelial cells, which are the main target cells, primarily depend on the RIG-I pathway for the induction of interferon (IFN) [81]. The intracellular identification of viral PAMPs prompts virus-infected cells to secrete type I interferons. These interferons then connect with the interferon α/β receptor on surrounding cells, leading to the activation of the interferon signaling mechanism. The activation of this signaling pathway leads to the expression of numerous interferon-stimulated genes (ISGs) and prompts the cell to enter an ’antiviral state,’ thereby restricting the virus’s ability to propagate further [81,82].

### 7.2. Insight into the Antiviral Responses Elicited by Type-1 Interferons (IFN-I)

As the standard immune response to viral attacks, the IFN-I pathway restricts a broad spectrum of viral agents by inducing the production of numerous interferon-stimulated genes (ISG). There are molecular elements in viruses that are conserved and missing from eukaryotic hosts. Recognized by immune sensors in the host, these molecular traits, termed pathogen-associated molecular patterns (PAMPs), initiate a response aimed at combating viral infections [81,82]. TLR7 and TLR9, situated in the endosome, function as viral sensors that specifically recognize viral RNA and dsDNA. In the cytoplasmic environment, there are also viral sensing mechanisms like RIG-Like-Receptors (RLR) that can detect RNA associated with viruses, in addition to cGAS and IFI16 receptors that are attuned to viral DNA. Immune sensors respond to the presence of viral nucleic acids by initiating a specific signaling cascade that activates transcription factors IRF3 and IRF7, as well as NFkB, resulting in the production of type-1 interferons, such as IFNα and IFNβ [82,83]. These interferons are secreted outside the cells and communicate signals either in a paracrine or autocrine manner. When IFN-I binds to the IFNAR complex on cell membranes, it activates the signaling cascade linked to IFN. Initially, Janus Kinase proteins (JAK) are activated, causing the phosphorylation of STAT family proteins, namely STAT1 and STAT2. This action prompts the dimerization of STAT proteins and their subsequent relocation to the nucleus. Thus, this leads to their engagement with IRF9, facilitating the transcriptional activation of a significant number of interferon-stimulated genes (ISGs). The comprehensive spectrum of ISGs includes factors that modulate the response elicited by interferon, with numerous ISGs acting as antiviral agents that can inhibit specific stages of the virus replication cycle [80,81].

### 7.3. How Viruses Undermine the Immune System’s Response to Interferon

The hostile cellular conditions instigated by type-1 interferon in response to viral threats have driven viruses to evolve a range of strategies aimed at circumventing this protective host response. The approaches can be divided into three main categories: blocking the production of IFN, obstructing IFN signaling pathways, and hindering the action of ISG effectors [83,84]. The activation of interferon synthesis can be broken down into three key components: the identification of viral RNA or DNA by viral detection mechanisms, the downstream signaling facilitated by adaptor proteins, including MYD88, MAVS, and STING, and the final step involving the activation of transcription factors IRF3/7 and NFκB. Pathogens evade the immune system’s surveillance by employing viral proteins that interfere with a specific phase of the interferon production process [84,85]. As an example, the NS1 protein found in the influenza virus hinders the ubiquitination of the RIG-I RNA sensor. The MYD88 adaptor, which plays a role in TLR signaling, is directly targeted and suppressed by the NS5A protein from the Hepatitis C virus. KSHV vIRF1 acts as an antagonist to the STING adaptor protein involved in RLR signaling. The IRF3 transcription factor, crucial for IFN production, is frequently hindered by viruses like HPV E6 and Herpes Simplex virus ICP0, which stop it from entering the nucleus [84,86].

Besides their role in thwarting viral detection and interferon production, viruses are also known to inhibit the JAK-STAT-IRF9 signaling pathway that mediates IFN responses, thereby preventing the activation of genes stimulated by interferon. In this pathway, the STAT proteins are essential and are among the most frequently attacked protein families by viruses through a variety of methods [86,87,88]. Dengue virus NS5 and parainfluenza virus V protein promote the degradation of STAT1 and STAT2, while Epstein–Barr virus LMP-1 and Marburg virus VP40 inhibit STAT phosphorylation. Additionally, adenovirus E1A and HPV E7 prevent the nuclear localization of the STAT-IRF9 complex. Effective IFN signaling prompts the cell to enter an antiviral phase, where numerous proteins are produced to inhibit virus-specific features that do not belong to the host. The MX1 and MX2 proteins, known for their role as ISG effectors, are associated with myxovirus resistance [82,87,88]. The presence of MX1 obstructs the endocytic movement of incoming viral particles, ensuring they do not reach their target within the cell. The protein MX2 is identified as a binding agent for the HIV-1 pre-integration complex, effectively blocking the virus from entering the nucleus and integrating into the host’s genetic material. Other significant proteins that act as ISG effectors consist of the OAS family, Protein Kinase R (PKR), and the Interferon-induced transmembrane (IFITM) proteins. The recognition of viral RNA by OAS proteins leads to their degradation via the activation of RNaseL. The presence of IFITM within the viral particles inhibits the fusion of viruses with host cells. IFITM proteins play a direct role in disrupting viral fusion when embedded within virions, but their primary antiviral activity occurs at the host cell membrane, where they restrict viral entry by modifying membrane fluidity and preventing the fusion of viral and endosomal membranes. Recent research shows that IFITM proteins can also integrate into virions during the budding process, particularly in the case of enveloped viruses such as influenza A virus (IAV) and HIV-1. This incorporation can impact the infectivity of the virus, with some evidence suggesting that virions containing IFITM proteins exhibit reduced fusion capacity. However, viruses have developed countermeasures to neutralize these effects, such as altering their lipid composition or interacting with specific viral proteins to circumvent IFITM-mediated restrictions. Consequently, while IFITM incorporation into virions may modulate infectivity, its primary antiviral function resides in host cells. Moreover, viral adaptations can potentially offset the restrictive impact of these proteins within virions [80,81,87].

## 8. Exploring Host–Virus Pathways for Enhanced Antiviral Treatments

### 8.1. Viral Transcription, Translation, and Host Factors

A significant number of the host factors currently known to impede IAV replication primarily focus on the stages of genomic replication and translation within the virus’s life cycle. Almost all vertebrates produce Myxovirus resistance (Mx) proteins, which are renowned for their ability to combat viral infections. Human Mx1 proteins exhibit significant antiviral properties against influenza A virus (IAV) by targeting the viral ribonucleoproteins (vRNPs) using two distinct approaches. The entry of vRNPs into the nucleus is obstructed, resulting in their accumulation within the cytoplasmic region. Furthermore, the conversion of cRNA into vRNA is impeded, potentially as a result of the viral NP and PB2 being sequestered [85,89]. Sensitivity to Mx1 in viruses is largely determined by the viral NP, which acts as the principal structural component of the vRNP. Activated by the presence of double-stranded RNA, protein kinase R (PKR) is a kinase that responds to interferon stimulation and demonstrates significant antiviral effects against multiple viruses, including IAV. Activation of PKR leads to its self-phosphorylation and the phosphorylation of vital substrates, including eIF-2α and IκB. When eIF-2α undergoes phosphorylation, it significantly disrupts the production of viral proteins, leading to a reduction in viral replication. The enzyme PKR also modifies IκB through phosphorylation, which in turn activates NFκB, facilitating the upregulation of genes associated with IFN [89,90].

The 2′, 5′-oligoadenylate synthetases (OAS), a family of genes stimulated by interferons, create 2-5A that leads to RNA degradation through the activation of RNaseL. When OAS1, OAS2, and OAS3 attach to double-stranded RNA, they are triggered to activate and start producing 2-5A from ATP. After being synthesized by OAS, 2-5A connects with RNaseL in the cytoplasm, triggering its dimerization and activation process. When RNaseL becomes activated, it is capable of degrading single-stranded RNA from both viral and cellular origins, which plays a crucial role in restricting viral replication and inducing apoptosis in the infected host cells. Also, evidence suggests that these degraded materials can initiate RIG-I activation, further amplifying the interferon response [91]. Humans possess four distinct proteins that are part of the IFN-induced protein family, which are defined by the presence of tetratricopeptide repeats. The ability of these proteins to attach to viral RNA plays a crucial role in obstructing the start of translation. Also, they engage with uncapped viral RNA and are capable of sequestering viral proteins and RNA within the cytoplasmic area. While the exact roles of these functions in IAV infection are still to be elucidated, it has been confirmed that IFIT proteins directly associate with the removal of IFIT3, IFIT2, IFT1, and IAV RNA results in a boost in IAV replication [90,91]. Certain host factors that combat viruses work by attaching themselves to viral proteins, which prevents those proteins from functioning properly. The host factor known as Moloney leukemia virus 10 (MOV10) is stimulated by interferon (IFN) and binds to the influenza A virus (IAV) nucleoprotein (NP), blocking its connection with importin-α. This action causes NP to be sequestered in the cytoplasm, thereby stopping the creation of the vRNP complex [92].

The interaction between DDX21 RNA helicase and PB1 hinders the creation of viral RNA complexes, causing a decline in the generation of both viral RNA and proteins. NS1 specifically targets DDX21 to hinder its antiviral capabilities and facilitates its engagement with various viral proteins, thereby enhancing viral replication. Another host element, Plakophilin 2 (PKP2), competes with PB2 for its connection to PB1, which ultimately leads to a decrease in the role of the IAV RdRp during its activity. The immunophilin superfamily includes Cyclophilin A (CypA), which has been shown to associate alongside the M1 protein associated with IAV [90,93]. Lately, efforts have been focused on hastening the breakdown of M1 through the ubiquitin proteasome 2 mechanism. CypE, another family member, interacts with IAV NP, hindering the self-association of NP and its connections with PB1 and PB2. The antiviral protein known as zinc finger antiviral protein (ZAP) is a host factor that can be induced by interferons, featuring two isoforms produced through alternative splicing, the long isoform (ZAPL) and the short isoform (ZAPS), which differ in their C-terminal ends. The downregulation of viral mRNA by ZAPS restricts the translation of NA, PB2, and PA [92,93,94]. The protein ISG15, which is induced by IFN and resembles ubiquitin, links to target proteins through UbcH8, Herc5, and UbE1L. Over 80 tripartite motif (TRIM) proteins act as antiviral host elements, involved in adaptive and innate immune mechanisms [94]. Numerous constituents of the TRIM family influence the signaling pathways activated by PRR in response to viral infections. TRIM22 serves as an antiviral factor by facilitating the polyubiquitination of IAV NP, which results in its breakdown via the proteasome. Furthermore, TRIM32 has the ability to attach to viral PB1 and mark it for degradation by ubiquitination, leading to its breakdown by the proteasome [94].

### 8.2. Block Assembly and Viral Release by Host Factors

Studies have shown that different cellular factors significantly affect the later phases of the replication cycle of the IAV. Evidence suggests that Cyclin D3, a vital component in cell cycle regulation, shows significant antiviral activity against the IAV. D3 overexpression ostensibly binds to viral M2, disrupting M1-M2 interactions essential for viral progeny assembly. An IFN-induced protein, BST-2, inhibits the release of IAV from infected cell membranes. BST-2, functioning as a transmembrane protein, has the ability to attach to enveloped viruses such as IAV at the membrane of host cells; however, its effectiveness in inhibiting these viruses varies with different strains of IAV [92]. Viperin, an interferon-inducible protein associated with the endoplasmic reticulum, plays a role in suppressing viral replication through various mechanisms. Viperin inhibits isoprenoid production, interfering with the lipid rafts that IAV relies on for budding from the membrane of plasma. Plasminogen activator inhibitor 1 (PAI-1) is an interferon-stimulated gene that restricts the spread of influenza A virus by blocking the airway proteases needed to activate the virus’s hemagglutinin glycoprotein, crucial for its maturation. The presence of genetic polymorphisms in the SERPINE1 gene, which can cause either a total or partial reduction in PAI-1 in humans, is associated with a greater vulnerability to IAV in vitro [92].

## 9. Novel Strategies in Managing Chronic Viral Infections

### 9.1. Focusing on Interactions Between Viral and Host Proteins for Treatment Options

By creating interactions with host proteins, viruses effectively hijack cellular functions necessary for entry, replication, translation, and release, relying on specific receptors and factors. Focusing on and interrupting these connections has become a hopeful strategy for creating treatments for viral diseases. It is noteworthy that a large percentage of antiviral medications that have received approval are classified as small-molecule inhibitors. Due to their relatively small size, they can easily penetrate cells and interact precisely with specific binding areas. The ease of taking medication orally emphasizes their effectiveness as a treatment option. Identifying the viral proteins vital to the life cycle of the virus marks the starting point for the development of small molecule inhibitors [95,96]. A key element of this procedure involves obtaining structural data that are crucial for creating medications specifically aimed at viral proteins. Methods for determining protein configurations in the development of antiviral medications encompass virtual screening facilitated by computers and high-throughput screening based on experimental techniques. Techniques for computational drug discovery, including virtual screening methods that utilize X-ray crystallography, homology modeling, and cryo-electron microscopy, produce a variety of protein structures [95].

### 9.2. A Novel Family of Host Factors That Combat Viruses Is Histone Deacetylases

The scientific community has recently shown heightened curiosity regarding histone deacetylases (HDACs), a type of cellular enzyme, due to their increasingly recognized contributions to several human health issues, cardiovascular complications, neurodegenerative conditions, encompassing cancer, and other diseases. Remarkably, there is a rising amount of data supporting the significance of HDACs in the context of viral infections, with numerous investigations demonstrating their dual capacity to either aid or obstruct these infections. Some research has demonstrated that HDACs 1, 2, 6, and 11 contribute to antiviral defense during IAV infection and that IAV targets these enzymes to diminish their expression, likely to weaken their antiviral capabilities [97].

### 9.3. Conventional Functions of Histone Deacetylases

HDACs primarily serve to counteract the actions of HATs, playing an essential role in preserving the balance of protein acetylation and deacetylation. Depleting acetyl groups from histone and non-histone proteins can affect the shape of chromatin or the performance of transcription factors, leading to marked changes in the expression of genes. Hence, the molecular modifications triggered by HDACs can significantly alter cellular functions, which in turn are essential for understanding health and disease [97].

### 9.4. Post-Translational Modifications and HDACs

Various post-translational modifications, including ubiquitination, sumoylation, methylation, neddylation, acetylation, propionylation, crotonylation, and butyrylation, can be added to the ε-amino group of lysine. Thus, the process of acetylating the ε-amino group inhibits any other changes from being made to that particular residue. Through this approach, HDACs play a secondary role in enhancing the regulation of different modifications that can dramatically affect how a protein operates within the cellular environment. Acetylation, for instance, is recognized to obstruct the process of ubiquitination, potentially resulting in the degradation of the tagged protein by the proteasome [98,99]. Therefore, HDACs can speed up the destruction of proteins by uncovering the ε-amino group, allowing for ubiquitination to occur. HDACs play a crucial role in the interaction between histones and chromatin by influencing the processes of histone acetylation and methylation. The process of adding an acetyl group to lysine 9 of histone 3 (H3K9) blocks methylation at that site and facilitates methylation at lysine 4 (H3K4). Following this, the synergy of these modifications allows the chromatin architecture to be more readily accessible, which aids in the activation of transcription. Thus, HDACs that act on H3K9 by removing acetyl groups obstruct the methylation process of H3K4, which in turn inhibits transcription [98,99,100].

### 9.5. Gene Transcription Control and HDACs

The conventional perspective on acetylation’s role in controlling transcription suggests that the acetylation of core histones diminishes DNA binding, thereby enhancing the accessibility of DNA for transcription processes. On the other hand, the removal of acetyl groups from histones enhances the binding between histones and DNA, which leads to a decrease in transcription activity. Recent studies are revealing that the processes of acetylation and deacetylation create a unique surface for protein attachment. To put it differently, the process of adding acetyl groups to histones could promote connections with transcriptional activators, whereas removing these acetyl groups may lead to the formation of binding sites for transcriptional repressors [100,101]. A recent investigation into HDAC5 and HDAC7 has revealed that HDACs can inhibit transcription without relying on their deacetylase function, which is quite intriguing. Moreover, recent studies indicate that the particular lysine residue that is deacetylated by HDACs might influence transcriptional repression. The removal of acetyl groups from H4K16 may cause a broad decrease in transcription, while the deacetylation of H4K12, H4K8, or H4K5 alone has little influence, but their combined effect is substantial. Increasing research points to the possibility that HDACs do more than just suppress transcription; they may also facilitate the activation of particular genes. Evidence for this can be seen in cells obtained from mice lacking Hdac3, which exhibit both increases and decreases in gene expression levels [102]. Similar levels of gene upregulation were observed in cells treated with HDAC inhibitors and in Hdac3 knockout cells. HDACs might inhibit the expression of transcriptional repressors, which could lead to an increase in gene expression indirectly. It is also conceivable that HDACs could remove acetyl groups, thereby enhancing the function of transcriptional activators or, conversely, suppressing the action of transcriptional repressors without altering histone modifications. Regardless, it is clear that HDACs serve multiple functions in the regulation of gene transcription, though the specific mechanisms underlying these roles have yet to be identified [102].

### 9.6. Connection of Health and Disease with HDACs

The substantial impact of HDACs on gene regulation and their ability to modify protein activity through non-histone deacetylation explains why they are linked to a wide range of health conditions and diseases in humans. HDACs are especially important due to their involvement in various processes, including development, cancer, neurodegenerative illnesses, immune-related issues, cardiovascular conditions, and respiratory diseases [101].

## 10. Conclusions

Chronic viral infections, including HIV, HCV, and HBV, highlight the complex interactions between viral escape mechanisms and the host’s immune system, ultimately leading to persistent illnesses. These viruses manipulate host cellular processes to evade immune detection, promote immune exhaustion, and disrupt cytokine signaling, enabling their long-term survival. The genetic variability among hosts, particularly differences in HLA alleles, adds another layer of complexity to immune responses and influences disease progression. Innovative host-targeted therapies that aim to regulate host factors critical for viral replication and immune function are emerging as a promising area in antiviral treatment, potentially addressing the challenges posed by traditional antiviral methods.

Future therapeutic interventions for chronic viral infections will likely focus on host-directed therapies (HDTs) that focus on critical host factors necessary for viral replication. Progress in functional genomics and proteomics has uncovered new host dependency factors, enabling the development of targeted approaches that reduce the risk of resistance while boosting antiviral effectiveness. Promising approaches include small molecule inhibitors, immune checkpoint blockades, and advanced immune cell therapies like CAR-T cells, which are designed to enhance viral eradication. Complementary strategies such as cytokine-based treatments and next-generation therapeutic vaccines aim to strengthen the host’s immune defenses against long-term viral infections. By combining these innovative methods, researchers can create more effective and durable treatment strategies, overcoming limitations associated with traditional antiviral treatments.

## Figures and Tables

**Table 1:** The host factors that contribute to the viral entry, replication, and secretion of HBV, HCV, and HIV.

Stage	HBV	HCV	HIV
Entry	NTCP (Sodium taurocholate cotransporting polypeptide)	CD81, Claudin-1, Occludin, SR-BI	CD4, CCR5, CXCR4
Replication	Host polymerases, m6A methylation	miR-122, lipid metabolism factors	NF-κB, Sp1, Tat protein interaction with host factors
Secretion	Endoplasmic reticulum (ER), Golgi apparatus	ER, Golgi, exosomal pathways	ESCRT (Endosomal Sorting Complex Required for Transport)
Immune Evasion	Downregulation of HLA-DP, HLA-DQ, IL-10 modulation	IFN pathway inhibition, suppression of ISG responses	Nef-mediated MHC-I downregulation, PD-1 upregulation
